# Genomics of a revived breed: Case study of the Belgian campine cattle

**DOI:** 10.1371/journal.pone.0175916

**Published:** 2017-04-20

**Authors:** Liesbeth François, Katrien Wijnrocx, Frédéric G. Colinet, Nicolas Gengler, Bettine Hulsegge, Jack J. Windig, Nadine Buys, Steven Janssens

**Affiliations:** 1 Department of Biosystems, Research group of Livestock Genetics, KU Leuven, Leuven, Belgium; 2 Gembloux Agro-Bio Tech, Agriculture, Bio-engineering, and Chemistry Department, University of Liege, Gembloux, Belgium; 3 Centre for Genetic Resources, the Netherlands (CGN), Wageningen University & Research, AH Wageningen, The Netherlands; 4 Animal Breeding and Genomics Centre, Wageningen University & Research, AH Wageningen, The Netherlands; National Cheng Kung University, TAIWAN

## Abstract

Through centuries of both natural and artificial selection, a variety of local cattle populations arose with highly specific phenotypes. However, the intensification and expansion of scale in animal production systems led to the predominance of a few highly productive cattle breeds. The loss of local populations is often considered irreversible and with them specific qualities and rare variants could be lost as well. Over these last years, the interest in these local breeds has increased again leading to increasing efforts to conserve these breeds or even revive lost populations, e.g. through the use of crosses with similar breeds. However, the remaining populations are expected to contain crossbred individuals resulting from introgressions. They are likely to carry exogenous genes that affect the breed’s authenticity on a genomic level. Using the revived Campine breed as a case study, 289 individuals registered as purebreds were genotyped on the Illumina BovineSNP50. In addition, genomic information on the Illumina BovineHD and Illumina BovineSNP50 of ten breeds was available to assess the current population structure, genetic diversity, and introgression with phenotypically similar and/or historically related breeds. Introgression with Holstein and beef cattle genotypes was limited to only a few farms. While the current population shows a substantial amount of within-breed variation, the majority of genotypes can be separated from other breeds in the study, supporting the re-establishment of the Campine breed. The majority of the population is genetically close to the Deep Red (NL), Improved Red (NL) and Eastern Belgium Red and White (BE) cattle, breeds known for their historical ties to the Campine breed. This would support an open herdbook policy, thereby increasing the population size and consequently providing a more secure future for the breed.

## Introduction

After the domestication of wild aurochs (*Bovis primigenius*), the populations of both taurine (*Bovis taurus*) and zebu (*Bovis indicus*) cattle spread out over the world coinciding with human migration [1–3]. Gradually, local populations with specific and highly adapted phenotypes emerged as a consequence of both natural and artificial selection [4,5]. Over the last decades, intensification and expansion of scale in livestock farming has led to the demise of local populations more suitable to extensive farming systems [4,6]. While selection within local populations was attempted, the lower attained selection intensity and lesser focus on breeding strategies, continuously increased the gap with highly productive breeds [7]. Consequently, local breeds are forced out of the (current) market; however, qualities like robustness (i.e. the ability of an animal to function in a broader range of environments) and adaptability are likely to be preserved in these local populations [4,8,9].

In Belgium, the robust constitution of the Campine breed and its ability to produce on a meager diet was well known [10,11]. The breed belonged to the group of European lowland red and blue pied breeds, also present in Western Germany, part of the Netherlands, and Luxembourg [10–12]. To improve the sub-optimal production of all Belgian cattle populations, Shorthorn cattle was introgressed starting from 1845. However, the results from most crosses were below expectations and the proportion of Shorthorn genetic material in Campine cattle and other local breeds was gradually reduced in subsequent generations. The following decades, breeding animals from Meuse-Rhine-Yssel (MRY) cattle were imported but also within-breed selection was performed to improve the Campine breed [10,11,13].

In 1954, Belgium was divided in 6 cattle breed zones, each of them maintaining one cattle breed. The Campine breeding zone is covered by the northern provinces (Antwerp, Limburg) and, in addition, the Belgium’s Eastern Cantons with a population of Eastern Belgium Red and White (EBRW) cattle [11]. After the dissolution of the breed zones in 1972, the Campine breed was absorbed into the Belgian red pied breed along with Flemish Red and Flemish Red and White [11]. Quickly Campine bulls were replaced by Dutch MRY bulls, and from the 1980s onwards, by Holstein cattle, leading to the Belgian Red Holstein population [11,12].

From this point on, only a few isolated farmers maintained animals from the original Campine population. However, as no officially registered Campine bulls were available, these breeders were forced to use bulls from phenotypically similar breeds such as MRY and Deep Red (NL) as well as other red-pied breeds. In 2012, the herdbook of the Campine breed was re-established and currently, efforts are made to revive this local breed.

Due to the absence of an official herdbook from 1972 onwards and the subsequent import of foreign genetic material, the current Campine population could be a “counterfeit” population as stated in Felius et al. [14], phenotypically similar but genetically different from the original population. The objective of this study is to assess the effect the absence of an official herdbook has on a local population using genomic information. Additionally, we investigated whether we can still consider the current Campine population as a genuine breed and whether it is still worthwhile to conserve its diversity?

## Material and methods

### Samples

A total of 346 Campine animals were sampled from ten farms in Flanders, all registered at Coöperatie Rundveeverbetering B.V. (CRV) at the moment of sampling. Venous blood was collected by the farm veterinarian in 3 ml or 9 ml collection tubes (Heparin or EDTA), cooled on ice for transport and frozen (-20°C) upon arrival. Farmers gave permission for their animals to be used in the study and the study was approved by the Ethical Committee for Laboratory Experimentation (ECD) (KU Leuven) [P089/2014]. In addition, semen straws of Rouge des Prés (formerly known as “Maine Anjou” (n = 11)), Flemish Red (n = 28), and Red Holstein (n = 20) were obtained from CRV and Association Wallonne de l’Elevage (AWE). Other samples were obtained from the Centre for Genetic Reserouces, the Netherlands (CGN) of Wageningen University & Research (53 hair samples of Deep Red, 2 hair samples of MRY, and 6 semen straws of Improved Red).

### DNA extractions, genotyping, and quality control

For the Campine breed, 289 samples of animals registered as purebred were selected for genotyping using following criteria; all (candidate) breeding bulls were included (22 animals) and all animals born before 2007 (n = 29). The remaining animals were chosen randomly but proportionally to farm size as no pedigree information was available (Table 1). This selection was done at two points in time as four farmers entered the new Campine herdbook only later on (farmers 1, 2, 5, and 8). DNA was extracted using the MagAttract DNA Blood Midi M48 kit (Qiagen). All samples from the Campine breed and additional breeds were genotyped on the Illumina^®^ BovineSNP50 Genotyping Beadchip containing 54,609 SNPs distributed across the genome. Additionally, genotypes of MRY (149 animals) on the Illumina^®^ BovineHD Genotyping Beadchip were provided by Wageningen Livestock Research. Genotypes from the Illumina^®^ BovineSNP50 Genotyping BeadChip of Belgian Blue (BBL) and EBRW were obtained in collaboration with Gembloux Agro-Bio Tech and AWE (EBRW, 50 animals) and AWE (BBL, 50 animals). Additional genotypes from Improved Red (n = 12) were obtained in collaboration with Gembloux Agro-Bio Tech. Lastly, genotypes from the Illumina^®^ BovineSNP50 Genotyping for Black Holstein (61 animals) and Rouge des Prés (23 animals) were obtained from the study of Gautier et al. [15]. An overview of the number of samples per breed and their origin can be found in Table 2.

**Table 1:** Detailed description of the number of samples selected for the different analyses per farm.

Farmer	*n*^1^	Breeding objective^2^	*n* selected for analysis within Campine breed^3^	*n* selected for between breed analysis (total = 100)^4^
**1**	28	Dairy	8	3
**2**	48	Dairy	12	4
**3**	104	Dairy	81	28
**4**	48	Dual-purpose	24	8
**5**	214	Dairy	51	18
**6**	59	Beef	37	13
**7**	33	Dairy	17	6
**8**	9	Dual-purpose	5	2
**9**	8	Dairy	4	2
**10**	66	Dairy	45	16

^1^Number of animals present at the farm at time of sampling

^2^Breeding objective of the farm

^3^Number of animals selected for genotyping

^4^Number of genotyped individuals selected to be included in the within breed analysis

**Table 2:** Description of the number of samples available per breed and the source.

Breed	*n*^1^	Source
**Campine**	289	KU Leuven
**EBRW**	50	Gembloux Agro-Bio Tech, AWE
**Deep Red**	53	Centre for Genetic Resources (CGN)
**Improved Red**	18	Centre for Genetic Resources (CGN), Gembloux Agro-Bio Tech
**MRY**	151	Wageningen Livestock Research
**BBL**	50	AWE
**Rouges des Prés**	34	AWE, Gautier et al. [15]
**Flemish Red**	28	CRV
**Black Holstein**	61	Gautier et al. [15]
**Red Holstein**	20	CRV

^1^Number of genotyped individuals

Quality control was assessed in several ways using Plink v1.9 [16]. For the analysis within the Campine breed, SNPs with a minor allele frequency (MAF) < 1%, call-rate < 95%, and Hardy-Weinberg equilibrium q-value < 5% were discarded. A threshold for minimum call rate per individual was set to 95% and SNPs were pruned for linkage disequilibrium (LD) using a window of 50 SNPs, step size of 5, and VIF of 2 (—indep-pairwise). After quality control 284 individuals and 37,776 SNPs remained.

For the analysis between breeds, we first reduced the discrepancy in number of genotyped individuals among the breeds by reducing the dataset of the Campine breed (284 individuals) and MRY (149 individuals) to 100 individuals per breed. For the Campine breed, all breeding bulls were maintained and the remaining individuals were selected proportionally per farm (Table 1). In order to keep the full identity of the breed and of each farm, the genomic distance tree was used based on the genomic relationship matrix within each farm. The function *cutree* of the R Statistical package was used with *k* equal to the number of individuals selected per farm (Table 1) in combination with the random selection of one individual of each cluster [17]. Within the MRY population, the same procedure was used to select 100 individuals. Next, quality control was assessed using MAF < 1% and call-rate 95% were discarded. A threshold for minimum call rate per individual was set to 90%. After quality control 492 individuals and 29,245 SNPs remained for the analysis, this reduction in number of SNPs is the combination of different SNP arrays and the use of the genotypes available from Gautier et al. [15].

### Diversity and population structure

#### Inbreeding

The individual inbreeding coefficient was computed based on runs of homozygosity (ROH) using Plink v1.9 using a sliding window of 50 SNPs. The minimum number of SNPs to call a ROH (*l*) was calculated using the method proposed by Purfield et al. [18]: $l=\frac{{log}_{e}\;(\frac{\alpha }{n_{s}n_{i}})}{{log}_{e}(1-\bar{het})}$ with n_s_ the number of SNPs, n_i_ the number of individuals, and α the percentage of false positive ROHs (set to 0.05), resulting in a minimum of 45 SNPs per ROH. The minimum length of a ROH was set to 500kb to ROHs arising from LD. The maximum gap of 1000kb and a density of 1 SNP / 120kb. In addition only one missing SNP and no heterozygotes were allowed within the window [18,19].

#### Current effective population size

The current effective population size was estimated using the method developed by Weir et al. [20], adapted by Waples [21,22].

#### PCA and DAPC

To assess population structure within and between breeds, principal component analysis (PCA) was performed as implement in the R package *ade4*. Additionally, a discriminant analysis of principal components (DAPC) was performed for the between breed analysis using the R package *adegenet* to identify the complex between-group structure [17,23,24].

#### Clustering

Unsupervised K-means hierarchical clustering without prior knowledge was performed to assess the differentiation between three related populations; Campine, Deep Red, Improved Red, and EBRW (K = 4) using the “find.cluster” function in the R package *adegenet* [24]. In addition, the ancestry models implemented in FastSTRUCTURE were employed to assess the relation between these four breeds and the appropriate number of clusters was chosen using the “chooseK.py” using different choices of K [25]. To identify the genetic structure and differences between all breeds the program FastSTRUCTURE was used with default convergence threshold (10^−6^) for different runs of K (1–10). Afterwards, model complexity was assessed using the “chooseK.py” algorithm [25]. The results from FastSTRUCTURE were visualized with CLUMPAK [26].

## Results

### Diversity and population structure within the Campine population

**Fig 1:**
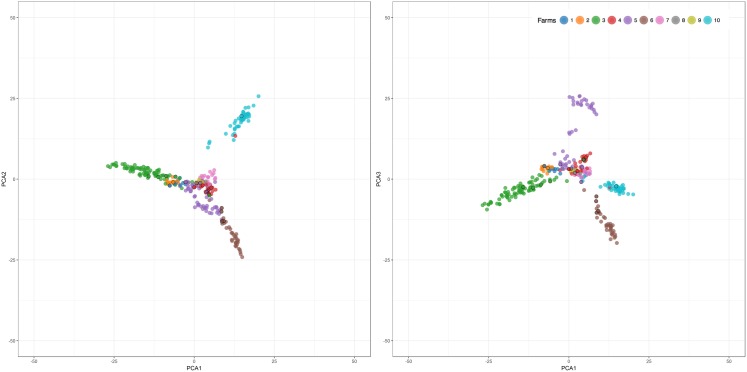
Principal component analysis within the Campine population indicating the position of the different farms on PC1, PC2 (a) and PC1, PC3 (b). The breeding bulls belonging to each farm are indicated using larger points.

To assess the diversity in the Campine population under study (284 individuals) the average inbreeding (*F*) and current effective population size (*N*_*e*_) were estimated at 4.1% and 81 individuals respectively. Using PCA the state of the within-population variation was visualized and several subgroups can be identified when considering PC1, PC2, and PC3 which explain 9.79% of the total variation (Fig 1). Contrary to the large amount of variation in this breed, the breeding bulls present in the population are more centrally located in the PCA analysis. Three subgroups can be assigned to different farms with a fourth central subgroup combining animals originating from the remaining farms; however, adding PC3 shows additional variability within this last group as well.

### Differentiation and admixture among ten related cattle breeds

**Fig 2:**
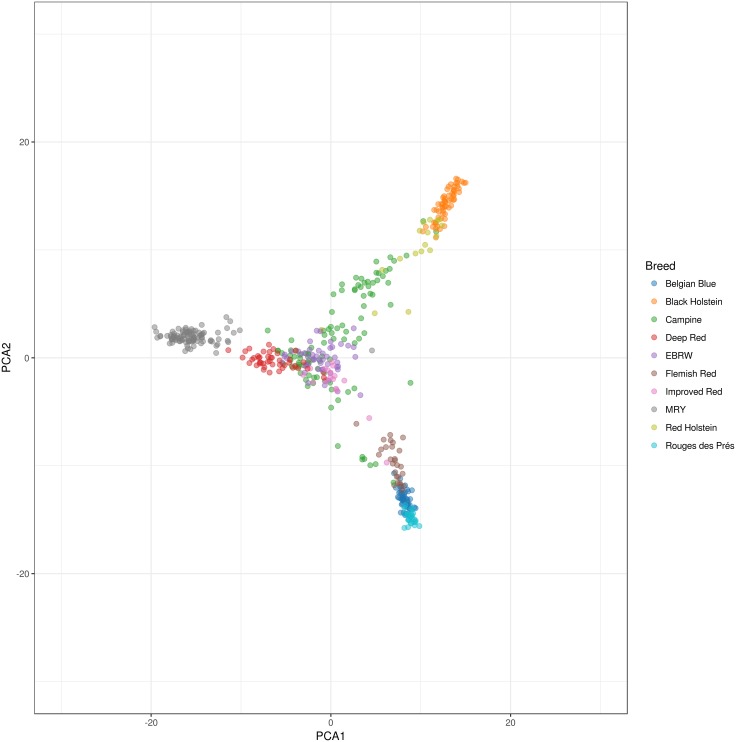
Principal component analysis showing the relation between the Campine population and nine additional breeds.

**Fig 3:**
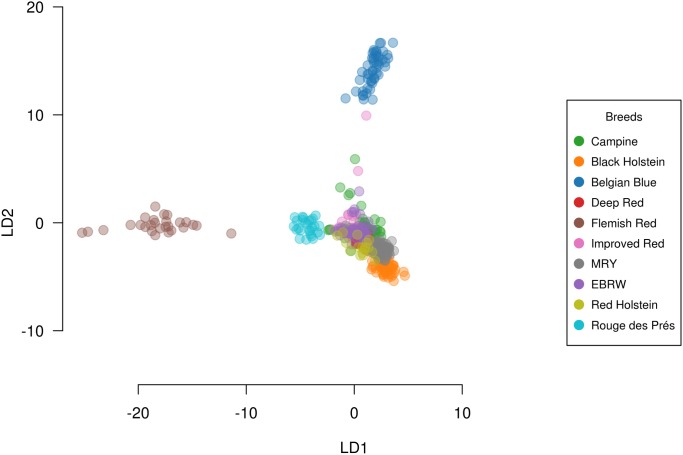
Discriminant analysis of principal components (DAPC) based on the between-breed analysis.

**Fig 4:**
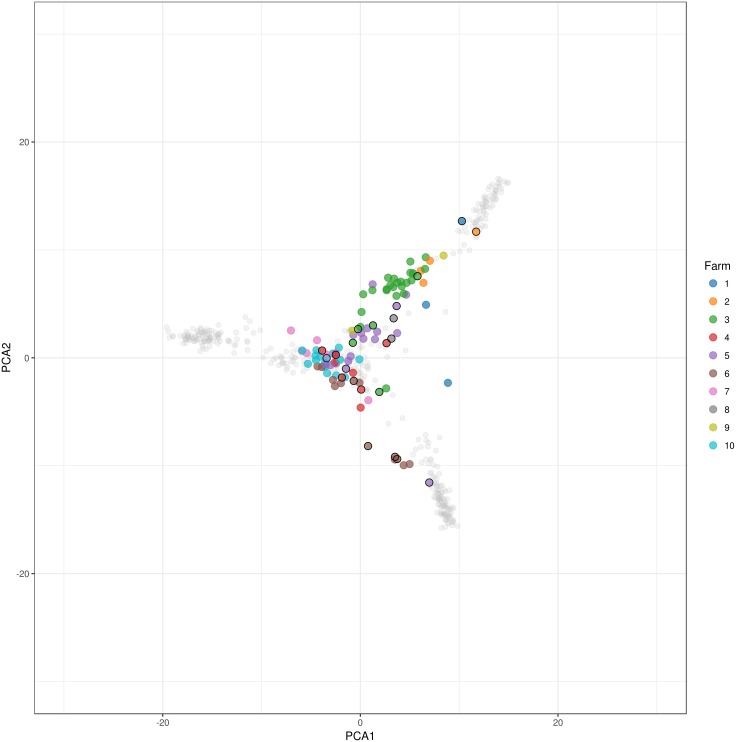
Principal component analysis of the between-breed analysis (similar to Fig 2) with emphasis on the Campine population (all other breeds colored in grey) and the position of each farm and respective breeding bulls (squares).

The variation between the ten related cattle populations was assessed using PCA as shown in Fig 2. The first three principal components explain 7.51% of the total variation. Most breeds under selection are clearly demarcated, such as Black Holstein, MRY, Rouge des Prés, and Belgian Blue. However, while most individuals of Red Holstein are close to Black Holstein population, some show a closer similarity to the local red (-pied) breeds. The Campine breed shows a large amount of variability, with one subgroup closer to the Holstein population and a smaller group towards the beef breeds. We can identify a central group from the Campine population is close to the Improved Red, Deep Red, and EBRW population. These results are corroborated by the DAPC analysis (Fig 3); however, this analysis also shows the close location of the Red and Black Holstein to the Campine population. In addition, including the information of the different breeds showed that while the breeding bulls are located more centrally on the within-breed PCA (Fig 1), they show more differentiation on the between-breed PCA (Fig 4). By comparison of Figs 2 and 4, four bulls are positioned close to the beef-type breeds (BBL and Rouge des Prés) while three bulls are close to the Holstein population. These bulls belong to farms with a strong influence of beef or dairy breeds respectively or possibly a focus on beef or milk production.

**Fig 5:**
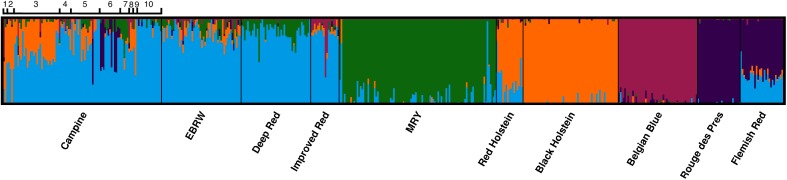
FastSTRUCTURE hierarchical clustering method for the between-breed analysis with additional information on position of each Campine farm (1–10) using K = 5.

**Fig 6:**
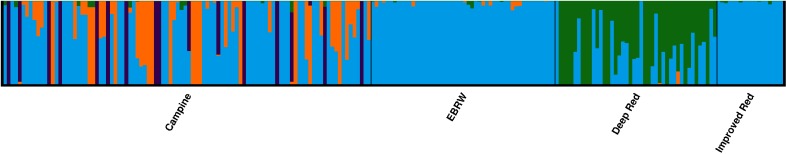
FastSTRUCTURE hierarchical clustering for the three historically related breeds: Campine, Deep Red, and EBRW using K = 4.

FastSTRUCTURE was used to assess the differentiation and genetic similarity of the different cattle populations (Fig 5). The optimal number of clusters to explain the structure of the data was K = 5. It clearly demonstrates the variability in the Campine population, where no clear identity can be identified. All local breeds (Campine, Deep Red, EBRW, and Improved Red) are clustered in one group and the same identity can be seen in Flemish Red, MRY, and Red Holstein to a lesser extent. The farms with an influence of dairy or dual-purpose type animals (farm 1, 2, 3, 5, 8, and 9) show similarity to the Holstein populations; however, they still present mixed identities. The beef-orientated farm 6 shows only a few individuals resembling the beef breeds under study or MRY. The more central farms from the PCA analysis (Fig 4), farms 4, 5, 6, 7, and 10, show a mixed identity similar to Deep Red, Improved Red, and EBRW. An additional FastSTRUCTURE analysis using only these four breeds using K = 4 as optimal number of clusters, showed that the populations of EBRW, Improved Red, and Campine are close together (Fig 6). The Deep Red population does share some of the same genetic structure, although it clusters separately for the main part. The Campine population also shows a lot of variability. Using unsupervised hierarchical clustering, the relationship between these four cattle breeds was analyzed in more detail (Table 3). Rather than attempting to identify the optimal number of subgroups present, we aimed to assess how genetically different these three related cattle breeds are. Using K = 4, individuals from Campine, Deep Red, Improved Red, and EBRW were assigned to four clusters. The majority of the Campine population (51 individuals) was allocated into a cluster together with some individuals from Deep Red and all individuals from Improved Red and EBRW. Of the three remaining clusters, one can be identified as the remaining animals of Deep Red while the other two can be identified as the dairy-type farms 1, 2, and 3 and as farm 10 respectively (Farms 3 and 10, Fig 1). In addition, the DAPC analysis shows the close relationship between these breeds and confirms their shared history, although the last generations have undergone some divergence (Fig 3).

**Table 3:** Unsupervised hierarchical clustering of three historically related cattle breeds (Campine, Deep Red, Improved Red, and EBRW).

Breed	Cluster 1	Cluster 2	Cluster 3	Cluster 4
Campine	16	0	51	33
Deep Red	0	35	9	0
EBRW	0	0	50	0
Improved Red	0	0	18	0

## Discussion

With the focus on intensification and selection, the maintenance and management of local populations has often been neglected [4]. Recently, the interest in local breeds has increased and, in some circumstances, attempts have been made to revive lost breeds [12]. However, the question remains how the accumulation of exogenous genes over generations of introgression has affected the breed’s authenticity on a genomic level [7]. The example of the Austrian Murbodner shows that once phenotypically similar breeds have been introgressed routinely, excluding these introgressed genotypes using only pedigree information and phenotypic assessments is impossible, leading to the loss of the genetic identity of a breed [27–29]. As a consequence, one might question the purpose of conserving such a population.

Using the revived Campine breed as a case study, we first assessed the effect of the absence of a herdbook on the current population and its genetic diversity. A herdbook provides a breeding objective as well as access to approved breeding bulls. Its absence forced farmers to use bulls from phenotypically similar breeds such as MRY, Deep Red, and Holstein among others (with the exception of one farm using own bulls). This led to a large amount of differentiation within the current population due to farm-specific definitions of a “Campine” animal. This subdivision across isolated farms did not lead to a decrease of the genetic diversity, on the contrary, a high level of overall diversity was conserved. The routine introgression from other breeds on some farms likely has added to this high level of diversity as well [30].

Despite this high diversity, the low *N*_*e*_ could reflects the bottleneck event that occurred after the breed’ cessation or it could point to the loss of LD phase due to more recent admixture events [30,31]. On the other hand, considering the 284 Campine animals under study, the low effective size is not below expectation as the ratio between effective and census population size has been reported to be around 0.2 over multiple species [32]. While it might be difficult to consider the current Campine breed as a clearly defined population due to farm-specific differences in selection objective, the high levels of genetic diversity within the breed offer opportunities for conservation.

Both historical as well as recent introgressions with phenotypically related breeds into the Campine breed are well known [10,11]. Especially the introgression of MRY and Deep Red cattle is substantial as is the introgression of Holstein, which was the preferred cross to improve the breed’s milk production [11]. Similarly to the Campine breed, the Austrian Murbodner was introgressed with Franken Gelbvieh and Fleckvieh to improve production and after herdbook closure in the 1970s, only a few breeders maintained the original breed [27]. However, unlike the Austrian Murbodner, the effect of the introgression of Holstein and beef breeds (BBL and Rouge des Prés) in the Campine population is limited to certain farms but did not influence the identity of the whole population under study. Similarly, in the Red and White Friesian cattle the within breed diversity was increased by introgression of Holstein in one farm, while animals from this farm did not contribute to overall diversity across breeds [33]. A more detailed analysis of the unique contribution of each breed analyzed in the current study is in preparation.

Combining the results from the DAPC and PCA analyses show that while the close proximity between the Campine breed and local breeds (Deep Red, Improved Red, and EBRW) is confirmed, the close proximity of Campine to both Holstein populations is caused by the introgression of Holstein genotypes in only a few Campine farms. Using FastSTRUCTURE several farms showed a strong resemblance to Red and Black Holstein. The Campine population carries an identity also found in Red Holstein; however, seems to have diverged from Holstein possibly through a specific selection objective. On the other hand, this Red Holstein population is known to be derived from the local red pied breeds through introgression with Black Holstein [11,12]. Our findings are similar to findings of the Franches-Montagnes horse breed, where the known introgression with Warmblood was limited to certain subpopulations [34]. In addition, the PCA analysis showed the central position of the majority of breeding bulls (n = 15), with the exclusion of some that are more orientated towards the dairy type (n = 3) and beef type (n = 4). The contribution of individuals with high levels of exogenous genes, especially breeding bulls, should be minimized with special attention to maintain low increase in inbreeding levels [29].

The Campine population presents a large amount of variation; however, the majority of animals are similar to Deep Red and EBRW. The exchange of breeding animals between these breeds and the Campine population has been well established [10,35,36]. Originating from one breeding group, they have diverged slightly from each other over the last generations presumably as a result of genetic drift [37]. The clear distance from the MRY population probably results from the additional selection in MRY as a dual-purpose breed whereas farmers aiming to maintain the old type of MRY cattle established the new herdbook of Deep Red cattle [11]. The Campine population shows an identity shared by Improved Red, Flemish Red, Deep Red, and EBRW and slightly in Red Holstein and MRY. Possibly this identity points to a common history between these breeds or past exchanges of breeding animals.

The relevance of reviving historical breeds using phenotypically similar but possibly genetically diverged breeds is under debate [27–29]. The statement of Felius et al. [14] that reviving the Campine breed has led to a counterfeit population containing (almost) none of its original characteristics does not seem to be supported with our results as the central group of Campine animals is clearly different from the Holstein population and still genetically close to the historically-related breeds of Deep Red and EBRW. It can be expected that at least a part of the original Campine diversity has been maintained as farmers were very attached to this breed. A substantial amount of introgression from phenotypically similar breeds is present in some farms; however, our results shows that Campine, Deep Red, and EBRW still contain identities unlike any of the other breeds. This seems to indicate that not all authentic genotypes are lost; contrary, the choice of breeding bulls can help establish a more clearly defined breed still representative of the former Campine population.

Although the diversity measures show no pressing issues, the presence of only few registered breeding bulls with original genotypes (15 animals) will most certainly pose problems in the near future. Additionally maintaining a closed breed policy might not be in the best interest of conservation [38]. The relationship between Deep Red, EBRW, and Campine has been well documented and they were considered as being one breed group [10]. This historical relationship is supported by our results. Felius et al. [14] rightly raises the question whether all breeds should be maintained. It may not be advisable to consider them as one population as crossbreeding would endanger the legitimacy of the products of a breed for conservation and establishment of a PDO (protected designation of origin) which is based on geographic location, know-how, and historical arguments [7,29,39]. In addition, defining a common breeding goal might prove difficult [40]. However, an open breeding policy would facilitate future selection and, in combination with the breed’s authenticity, provide a basis to obtain PDO products and create an economically viable production system to support the conservation of this breed [41]. Such an open herdbook policy is supported by clustering analysis, which was unable to distinguish between a substantial group of the Campine population, Deep Red, and EBRW cattle. In addition, both DAPC and FastSTRUCTURE analyses show a close relationship between these breeds. Genetic screening of breeding animals to ensure “genetic compatibility” should not endanger the breed’s authenticity or further reduce the frequency of possible rare haplotypes [37,42].

It is clear that the absence of a central herdbook had profound effects on the population structure of the Campine breed. However, by importing phenotypically similar bulls the diversity was maintained at sufficient levels and only four farms out of ten diverged greatly. Our results based on genomic information showed that the current Campine population still carries authentic genotypes that are likely similar to the original breed as it still has a separate identity from the introgressed breeds and no clear distinction with two historically related breeds, Deep Red, and EBRW can be found. It seems that the absence of selection led to the preservation of the breed’s authenticity supporting the conservation of this breed. As there are no pressing concerns in terms of genetic diversity, redirection of diverging farms can be facilitated using those breeding bulls that were found to have authentic genotypes. However, after creating a more uniform population, an open breed policy with Deep Red and EBRW and screening of breeding animals would ensure the future of all breeds while conserving their unique identity. The enlargement of the breeding pool would facilitate selection and open opportunities for these dual-purpose cattle to new niche markets of high value products with specific requirements for milk or meat quality and characteristics. This study showed the use of genomic information in identifying authentic genotypes in a revived breed with animals phenotypically similar to the original population. Comparing the revived population with historically related breeds allows for the identification of these authentic genotypes as well as securing the future of the breed under study.
